# Low erythrocyte catalase enzyme activity is correlated with high serum total homocysteine levels in tunisian patients with acute myocardial infarction

**DOI:** 10.1186/1746-1596-8-68

**Published:** 2013-04-30

**Authors:** Yosri Noichri, Abdelkader Chalghoum, Latifa Chkioua, Bruno Baudin, Samia Ernez, Salima Ferchichi, Abdelhédi Miled

**Affiliations:** 1 Biochemistry Laboratory CHU Farhat HACHED, Street Doctor Moreau, 4000 Sousse, Tunisia; 2 Department of Biochemistry, Hospital Saint-Antoine, 184 Street Faubourg Saint-Antoine, 75571 Paris Cedex 12, France; 3 Department of Cardiology, CHU Farhat Hached, Street Doctor Moreau, 4000 Sousse, Tunisia

**Keywords:** Catalase, Hyperhomocysteinaemia, Lipid peroxidation, Acute myocardial infarction

## Abstract

**Background:**

An imbalance between pro-oxidants and antioxidant systems has been suggested to be implicated in the physiopathology of acute myocardial infarction (AMI). We aimed to evaluate the antioxidant capacity in Tunisian patients and to assess the possible relationship between erythrocyte catalase enzyme activity and hyperhomocysteinaemia.

**Methods:**

108 patients with AMI and 81 healthy subjects were enrolled in this study. Catalase erythrocyte enzyme activity was determined spectrophotometrically whereas “total antioxidant status” (TAS) concentration was measured by a commercially available method. Serum total homocysteine (tHcy) level was determined by a fluorescence polarization immunoassay (FPIA). Lipid peroxidation was measured with a fluorimetric method as “thiobarbituric acid reactive substances” (TBARS).

**Results:**

Compared with healthy subjects, patients with AMI had significantly lower catalase activity (*P<0.001*), TAS concentrations (*P<0.001*), and significantly higher serum tHcy (*P<0.001*) and TBARS levels (*P<0.001*). Erythrocyte catalase enzyme activity was negatively correlated with serum tHcy and TBARS while serum tHcy and TBARS were in positive correlation. Furthermore, the unbalance between pro-oxidants and antioxidants seems to be more aggravated in patients with Q wave AMI compared to patients with non-Q wave AMI.

**Conclusion:**

Our results suggest the involvement of hyperhomocysteinaemia in the drop of erythrocyte catalase activity related to myocardial ischemia reperfusion. Hyperhomocysteinaemia may increase the myocardial wall dysfunction under ischemia reperfusion by excessive production of reactive oxygen species which is made evident by increased lipid peroxidation.

**Virtual slides:**

The virtual slide(s) for this article can be found here: http://www.diagnosticpathology.diagnomx.eu/vs/1623509866881834

## Background

Myocardial infarction is a major cause of morbidity and mortality worldwide. Recently, an increase in the incidence of coronary heart disease (CHD) has been recorded in Tunisian cardiovascular disease register. Among diabetes, hypertension, abdominal obesity and smoking, a family lipoprotein disorder such as a high level of serum apolipoprotein B (Apo B) or/and a lower level of serum apolipoprotein A-1 accounted for almost all the population attributable risk of AMI [1,2]. Similarly, hyperhomocysteinaemia related to nutritional or genetic factors, such as methylenetetrahydrofolate reductase, endothelial nitric oxide synthase genes or with low paraoxonase activity led to increased risk of CHD severity. It can induce sustained injury of arterial endothelial cells and proliferation of arterial smooth muscle cells [3].

Atherosclerotic plaques are the major cause of Myocardial infarction. Other unusual causes of MI can be related to myocardial necrosis, or Thiamine deficiency causing alterations in heart metabolism [4,5]. Coronary artery occlusion can result in a reduction in myocardial blood flow [6]. In fact, myocardial ischemia occurs when myocardial oxygen demand exceeds the oxygen supply. Reperfusion of the ischemic myocardium can restore, afterwards, the blood flow but sudden massive increase in oxygen supply can lead to additional myocardial cell dysfunction and cell necrosis. Excessive production of Reactive Oxygen Species (ROS) has been most importantly proposed to mediate ischemia reperfusion injury. These species are toxic and may cause potential biological damage to all cellular components [7]. This occurs when there is an overproduction of ROS on one side and a deficit or inadequate availability of antioxidant systems on the other [6]. ROS generation may induce irreversible myocardial cell necrosis or apoptosis through triggering DNA fragmentation and caspase activation and causing myocardial injury [8,9]. Exposure to ROS from a variety of sources led to develop a series of defense mechanisms to neutralize these species and so protect cells against their toxic effects. This is achieved mainly by enzymatic antioxidants such as catalase, or by non-enzymatic antioxidants such as vitamins. Catalase is believed to play a major role in the first line of enzymatic antioxidant defense. It is a tetrameric enzyme, consisting of four identical subunits that contain a single ferriprotoporphoryn group per subunit. Catalase reacts efficiently in peroxisomes with hydrogen peroxide (H202) to form water and molecular oxygen [10,11]. Hyperhomocysteinaemia and other cardiovascular risk factors have been suggested to be implicated in the imbalance aggravation between pro-oxidants and antioxidants linked to its pro-oxidant properties or in the impairment of antioxidant systems. The aim of our study is to evaluate the total antioxidant capacity and erythrocyte catalase activity in patients hospitalized for AMI. We aimed to access the possible relationships between erythrocyte catalase activity, hyperhomocysteinaemia and the severity of myocardial dysfunction under ischemia reperfusion.

## Materials and methods

### Study population

The study consisted of 108 patients with a mean age of 63 *±* 12 years admitted to the department of cardiology in Farhad Hached hospital in Tunisia. The diagnostic of AMI was established according to universal clinical criteria: chest pain which lasted for up 3 hours, ECG changes and serum troponin elevation. The control group consisted of 81 healthy volunteers with no history of coronary artery disease, diabetes, hypertension or inflammatory disease. Their mean of age was 59 *±* 9 years.

The electrocardiographic changes were recorded and assessed by 2 cardiologists who were unaware that the patients are included in our study.

Coronary risk factors have been referred in our study population according to universal definitions. Dyslipidemia was defined when total cholesterol concentration was ≥ 5,68 mmol/l, or triglyceride concentration ≥ 2,28 mmol/l. Hyperhomocysteinaemia was defined by a raised blood total homocysteine, exceeding 15 μmol/l.

Informed written consent was obtained from each patient and healthy subject according to the guidelines of our ethics’ committee.

### Blood collection and biochemical methods

Venous blood was collected after overnight fasting and within 24 hours after admission for chest pain. Serum was separated by centrifugation at 1500 g for 10 min, and then stored at −80°C until the day of analysis. Serum was used for the estimation of lipid peroxidation, total homocysteine (tHcy), apolipoproteins (Apo A-1 and Apo B) levels and total antioxidant status (TAS) concentration.

Total homocysteine in serum was measured by the “Abbott homocysteine assay”, a fully automated fluorescence polarization immunoassay (FPIA) from Abbott diagnostics. Serum apolipoproteins (Apo A-1 and Apo B) were measured by immunonephelometry (Dade Behring, Marburg, Germany). Plasma TAS was measured with commercial kit (Randox, Antrim, UK) according to the method of Miller et al. [12]. It can evaluate the total capacity of all antioxidants found in serum to neutralize the oxidative action of free radicals. Lipid peroxidation level was estimated by measurement of thiobarbituric acid reactive substances (TBARS) in serum according to the fluorimetric method of Yagi [13]. The pink chromogen produced by the reaction of thiobarbituric acid with lipid peroxidation products such as malondialdehyde (MDA) was estimated using 1,1,3,3-tetraethoxypropane as standard MDA. Serum TBARS levels were measured at 515 nm excitation and 553 nm emission.

Catalase activity was determined in washed red cells prepared immediately after sampling from whole blood anticoagulant with heparin. Then, cell lysates were stored frozen. Catalase activity was measured according to the spectrophotometric method of Goth [14]. This assay is based on the ability of hydrogen peroxide to form a stable stained complex with ammonium molybdate measured at 405 nm.

### Statistical analysis

Database management and statistical analyses were carried out using SPSS (Statistical Package for the Sociological Sciences), version 17.0. Results are presented as means ± SD, or percentages. Means were compared using Student for independent samples. The relations between variables were assessed with Pearson's correlation analysis. The significance threshold was set at 5% (p<0.05).

## Results

Clinical characteristics of patients with AMI and control groups are illustrated in Table 1. No difference was found between the 2 groups for the mean of age and sex. Among cardiovascular factors risk, diabetes, hypertension and dyslipidemia are greatly mentioned in patients compared with healthy subjects. Apolipoproteins disorder was observed in patients. There was a significant increase in serum Apo B level (*<0.001)* and a decreased serum Apo A-1 level (*<0.001)* compared with serum apolipoprotein levels in healthy subjects. In other hand, tHcy and TBARS, as a marker of lipid peroxidation, tended to be increased in patients whereas serum TAS concentrations and erythrocyte catalase activity were found lower than in controls.

**Table 1:** Clinical and biochemical features of patients with acute myocardial infarction and controls

	***Patients (n= 108)***	***Controls (n =81)***	***P***
*Age (years)*	63 *±* 12	59 *± 9*	*NS*
*Men (%)*	70	38	
*BMI (kg/m*^*2*^*)*	27 *± 6*	*23 ± 3*	*NS*
*Diabetes mellitus (%)*	61	0	
*Dyslipidemia (%)*	45	0	
*Hypertension (%)*	70	0	
*Smokers (%)*	50	6	
*Alcohol (%)*	25	0	
*Apo A1 (g/L)*	1.26 ± 0.19	1.5 ± 0.17	*<0.001*
*Apo B (g/L)*	1.29 ± 0.36	0.92 ± 0.22	*<0.001*
*TAS (mmol/L)*	1.47 ± 0.38	1.71 ± 0.22	*<0.001*
*Catalase (kU/g Hb)*	283 ± 141	652 ± 104	*<0.001*
*Homocysteine (μmol/L)*	26.9 ± 15.47	14.75 ± 2.69	*<0.001*
*TBARS (μmol/L)*	1.31 ± 0.36	0.71 ± 0.12	*<0.001*
*Troponine TnIc (μg/L)*	*5,04* ± 2,90	0	
Q wave AMI	9,13 ± 2,18		
Non Q wave AMI	4,09 ± 2,03		
*Treatment*			
*Statins* (%)	38	0	
*Beta-Blockers* (%)	40	0	
*Ca-Blockers* (%)	28	0	
*Diuretics* (%)	37	0	

Values are expressed as mean ± *SD* or percentage (%).

NS: not significant, BMI: body mass index, TAS: total antioxidant status.

We showed in Table 2 that erythrocyte catalase activity tended to decrease with smoking and/or arterial hypertension as cardiovascular risk factors. Serum tHcy level was significantly higher in hypertensive patients compared to the normotensive patients.

**Table 2:** Erythrocyte catalase activity and serum total homocysteine in patients with different cardiovascular risk factors

***Risk factor***		***Catalase (kU/g Hb)***	***P***	***Homocysteine (μmol/L)***	***P***
*Sex*	Men	294 ± 129	NS	25.8 ± 11.6	NS
	*Women*	271 ± 152		28 ± 18.4	
*Diabetes*	*Yes*	290 ± 152	NS	26.3 ± 15.9	NS
	*No*	268 ± 121		27.8 ± 14.9	
*Dyslipidemia*	*Yes*	282 ± 151	NS	26.5 ± 12.3	NS
	*No*	279 ± 124		27.6 ± 19.6	
Obesity	*Yes*	298 ± 131	NS	24.7 ± 13.2	NS
	*No*	268 ± 150		29.1 ± 17.2	
*Hypertension*	*Yes*	*211* ± 90	< 0.001	30 ± 17	< 0.001
	*No*	*412* ± 95		20.3 ± 8.2	
*Smoking*	*Yes*	*224* ± 105	< 0.001	28.4 ± 13.1	NS
	*No*	*377* ± 142		24.5 ± 18.7	
*Alcohol*	*Yes*	*275* ± 148	NS	24.5 ± 18.7	NS
	*No*	*387* ± 135		28.6 ± 11	

Values are expressed as mean ± SD.

NS: not significant.

### Erythrocyte catalase activity and serum tHcy level according to the severity of the myocardial dysfunction

**Figure 1:**
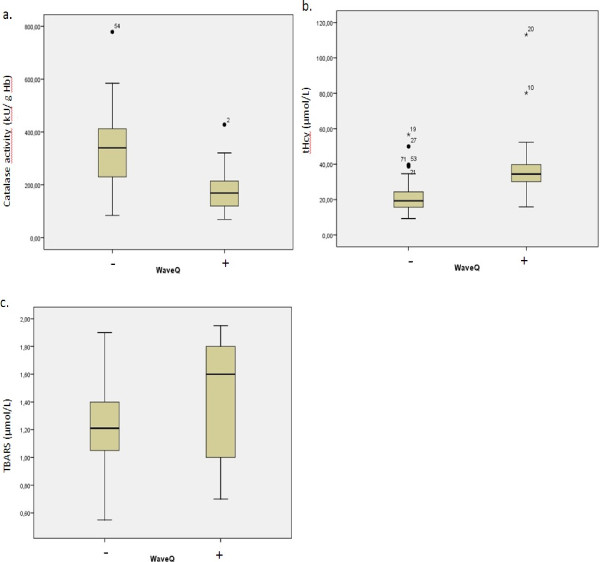
Comparison between patients with Q wave AMI and patients with non Q wave AMI according to serum total homocysteine level (tHcy) (p<0.001) (a), to erythrocyte catalase activity (p<0.001) (b) and to plasma TBARS level (p<0.05) (c).

Thirty one patients had a distinct increase of Q wave in the electrocardiogram established at the diagnostic. Patients with Q wave AMI had a significantly lower erythrocyte catalase activity, higher serum tHcy and TBARS levels compared to patients with non Q wave AMI (Figure 1).

### Results of Pearson correlation

**Figure 2:**
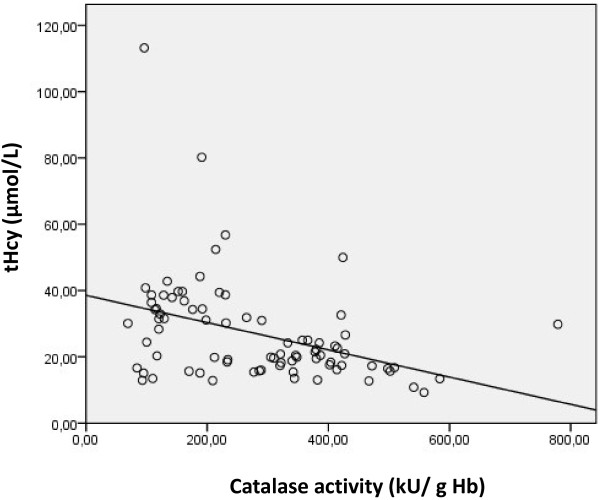
Correlation between erythrocyte catalase activity and serum total homocysteine in patients with AMI (r=-0.38, p<0.001).

**Figure 3:**
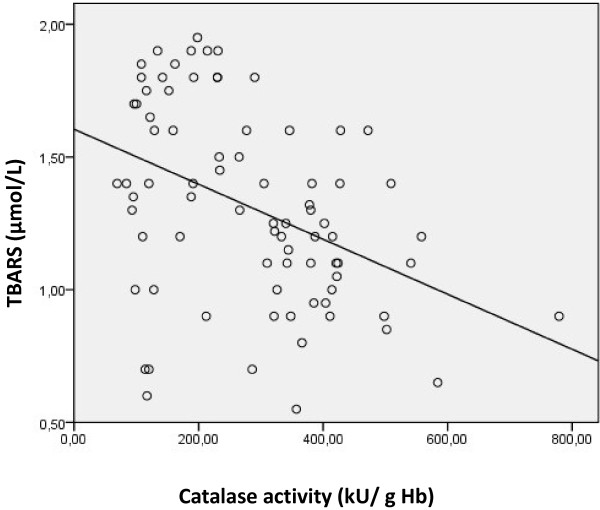
Correlation between erythrocyte catalase activity and TBARS levels in patients with AMI (r=-0.41, p<0.001).

**Figure 4:**
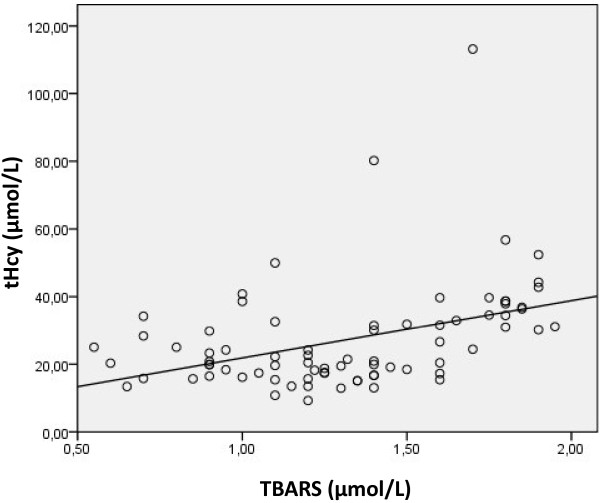
Correlation between serum total homocysteine (tHcy) and TBARS levels in patients with AMI (r= 0.4, p<0.001).

We studied the Pearson correlation between serum tHcy, erythrocyte catalase activity and serum TBARS in patients. We found that erythrocyte catalase activity is negatively correlated with both serum tHcy and TBARS (r=−0.38, p<0.001; r=−0.41, p<0.001) (Figures 2 and 3). Furthermore, serum tHcy was found positively correlated with serum TBARS level (r=0.4; p<0.001) (Figure 4).

## Discussion

Myocardial infarction is a complex and multifactor disease in which the cellular and molecular mechanisms contributing to myocardial injury need to be more defined. In our data, investigation about oxidative stress parameters shows an increased serum TBARS level, as a lipid peroxidation marker, and a drop in the total antioxidant capacity in patients with AMI compared to healthy subjects. According to our observation, Pasupathi et al. have considered that the increased serum TBARS is a consequence to excessive ROS generation. The authors reported it to the raised xanthine oxidase activity under ischemia reperfusion [15]. Other enzymatic and cellular sources are suggested to be implicated in ROS generation. Some of these are related to the uncoupling of mitochondrial electron transport in consequence to the lack of oxygen supply under ischemia, or to the impairment of NO synthase activity [7].

In our study, we observed a decrease in erythrocyte catalase activity in patients compared to controls. Accumulation of hydrogen peroxide under ischemia reperfusion can also inhibit antioxidant enzymes (high substrate inhibition) and alter its enzymatic conformation. According to Senthil et al., the decreased activity of catalase in patients with CHD could be due to the inactivation of this enzyme by cross linking or to the impairment of NO synthase. Nitrite oxide (NO) can bind reversibly to ferric iron, inhibiting afterwards catalase activity [16,17]. Erythrocyte catalase activity tended to decrease in patients with hypertension and smoking as the major cardiovascular risk factors. Cigarette smoke is an abundant source of free radicals. It contains more than 10^15^ free radicals including superoxide anion and NO. Chemical oxidants in cigarette smoke can cause the oxidation of DNA encoding for antioxidant proteins [18]. On the other hand, essential Hypertension is subjected to increased oxidative stress. It may damage the endothelium and impair endothelium-dependent vascular relaxation. ROS can act on angiotensin converting enzyme to increase its catalytic activity resulting in the increase in angiotensin II production, which, in turn, is a major endogenous inducer of NADPH oxidase. Oxidative stress, endothelial dysfunction and inflammation represent a key triad for the development and progression of Coronary heart disease [19,20].

The imbalance between pro-oxidants and antioxidants occurred under ischemia reperfusion can be aggravated by hyperhomocysteinaemia. The moderate hyperhomocysteinaemia revealed in patients can be reported to a hereditary defect of any of the Hcy metabolic enzymes (cystathionine β-synthase, methylene tetrahydrofolate reductase) or to the depletion in folic acid and vitamin B6 or B12 [3]. Increased serum tHcy may cause endothelial dysfunction by promoting free radicals. Homocysteine is readily oxidized. Its auto-oxidation is catalyzed by transition metal ions, such as copper leading to homocystine, homocysteine mixed disulfides and homocysteine thiolactone formation [21,22]. Homocysteine reduces the transition metal ion (M^n+^) to generate a thioyl radical (Hcy°). It is thought to react with homocysteine to generate a free radical intermediate that reduces oxygen to superoxide anion (O_2_°) and then to peroxide hydrogen generation [3,23]. In our data, we have shown a negative correlation between hyperhomocysteinaemia and erythrocyte catalase activity in patients with AMI. Homocysteine has the ability to bind proteins and to form disulphide bridges with cysteine residues within proteins. Milton N et al. suggest that modification of cysteine residues by Homocysteine may alter the enzymatic activity of catalase [24]. The excessive generation of ROS under ischemia reperfusion can affect the red cell metabolism and the possible hemolysis when more than 98% in blood catalase is located in erythrocytes [25]. Now this is in line with other works suggesting that Hcy can affect the antioxidant enzyme expression. Nanako et al. reported that homocysteine reduced the expression of superoxide dismutase (SOD) mRNA in cultured rat smooth muscle cells [26]. Other studies showed a positive correlation between plasma Hcy and genomic damage related to DNA hypomethylation which let us to suggest that Hcy can exert genotoxic effects on DNA genes encoding for antioxidant proteins [27].

Reactive oxygen species generated by Hcy auto-oxidation are involved through Fenton type reaction in lipid peroxidation. The serum tHcy and TBARS levels were found higher in patients’ with electrocardiogram presenting Q wave AMI compared to patients with non Q wave MI. Myocardial Infarction with Q wave can be used as a predictor of morbidity and mortality patterns after Myocardial events. Desmarais PL and al showed that individuals who had non-Q wave MI had better survival rates for the first 3 years after myocardial rehabilitation than did those who had Q wave MI [28]. Excessive lipid peroxidation can be directly involved in the myocardial necrosis manifesting as a myocardial wall dysfunction. Polyunsaturated fatty acids (PUFAs) such as arachidonic, linolenic and linoleic acids present the major targets for free radical attack. It has also been suggested that lipid peroxidation might proceed not only in plasma membranes but also in the nuclear membranes close to chromosomes, due to the loss of membrane integrity in cell membranes consisting of phospholipids. Lipid peroxidation products, such as 4-hydroxynonenal (HNE) and Malondialdehyde (MDA) are toxic. HNE leads to a decrease in protein thiols, disturbance of calcium homeostasis, inhibition of DNA, RNA and protein synthesis, inhibition of respiration and glycolysis [29]. Furthermore, HNE leads to mammalian cell death. It increases the expression of p53 family members as well as an increase of expression of the Bax pro-apoptotic gene [30]. MDA is able to interact with nucleic acid bases to form several different adducts and to exacerbate DNA oxidative damage, including genes encoding for antioxidant proteins such as catalase, glutathione peroxidase and SOD.

## Conclusion

Molecular mechanisms of myocardial necrosis under ischemia reperfusion need to be more defined. We have shown that hyperhomocysteinaemia, as a cardiovascular risk factor, may be involved in the drop of the enzymatic antioxidant activity. Hyperhomocysteinaemia can be involved in the excessive lipid peroxidation due to its pro-oxidant proprieties, aggravating the magnitude of the oxidative stress and the severity of the myocardial wall dysfunction. Further insights into the molecular mechanisms of the metabolic basis of hyperhomocysteinaemia will prove invaluable in the treatment and the prevention of cardiovascular diseases related to atherosclerosis.

## Abbreviations

AMI: Acute myocardial infarction; TAS: Total antioxidant status; tHcy: Total homocysteine; TBARS: Thiobarbituric acid reactive substances; ROS: Reactive oxygen species; ECG: Electrocardiogram; NO: Nitric oxide; SOD: Superoxide dismutase

## Competing interests

The authors declare that they have no competing interests.

## Authors’ contributions

All authors have done all the work in the laboratory. All authors have done the analysis of the results. All authors have given final approval of the version to be published. All authors read and approved the final manuscript.
